# Evaluation of a training programme for critical incident debrief facilitators

**DOI:** 10.1093/occmed/kqac125

**Published:** 2022-12-14

**Authors:** J Johnson, L Pointon, C Keyworth, N Wainwright, L Moores, J Bates, K Hinsby

**Affiliations:** School of Psychology, Lifton Place, University of Leeds, Leeds LS29JT, UK; Bradford Institute for Health Research, Bradford Royal Infirmary, Bradford BD96RJ, UK; School of Public Health and Community Medicine, University of New South Wales, Sydney, NSW 2033, Australia; School of Psychology, Lifton Place, University of Leeds, Leeds LS29JT, UK; School of Psychology, Lifton Place, University of Leeds, Leeds LS29JT, UK; Mid-Yorkshire Hospitals NHS Trust, Wakefield WF1 4DG, UK; Mid-Yorkshire Hospitals NHS Trust, Wakefield WF1 4DG, UK; Mid-Yorkshire Hospitals NHS Trust, Wakefield WF1 4DG, UK; Leeds and York Partnership NHS Foundation Trust, Leeds LS73JX, UK

## Abstract

**Background:**

Critical incident debriefs are a commonly used occupational health tool for supporting staff after traumatic work incidents. However, there is a dearth of literature evaluating training programmes for debrief facilitators.

**Aims:**

To evaluate a 5-day training programme to equip healthcare, social care and voluntary, community and social enterprise sector staff to act as post-incident peer supporters and debrief facilitators.

**Methods:**

A mixed-methods, single-arm, before-and-after study. Data were collected at baseline and post-training. The quantitative outcome measure was ‘Confidence’; the sum of two items measuring confidence in (i) supporting peers after critical incidents and (ii) facilitating post-incident structured team discussions. At post-training, quantitative and qualitative feedback regarding experiences and perceptions of the training was also gathered.

**Results:**

We recruited 45 participants between October 2021 and January 2022. Confidence in supporting peers following incidents and facilitating post-incident structured team discussions increased significantly following the training, *t*(35) = −6.77, *P* < 0.001. A majority of participants reported they would do things differently because of the training and that they found the training relevant, useful and engaging. Summative content analysis of qualitative feedback indicated that participants (i) believed the role plays were an important learning tool and (ii) thought it was important that the trainer was engaging. Some participants would have preferred in-person delivery.

**Conclusions:**

Participants valued training in post-incident peer support and debriefing skills. Organizations implementing post-incident support pathways could usefully include this training and ensure optimal uptake and engagement by (i) providing in-person and online delivery options and (ii) including role play as a learning technique.

## Teaser

Critical incident debriefs activate workers’ natural recovery mechanisms, but there is a lack of evidence regarding how debrief facilitators should be trained. This study conducted the first evaluation of a debrief facilitator training programme. The programme was associated with significant increases in participants’ confidence in facilitating debrief conversations and providing post-incident support. Respondents found the training useful, describing role plays as a challenging but essential learning device. Delivery by a personable, engaging facilitator was considered key.

**Key learning points**

**What is already known about this subject:**

- Critical incident debriefs are one long-standing technique which have been used in clinical and emergency settings to support staff following critical incidents.
- The most frequently highlighted recommendation for organizations looking to implement critical incident debriefs is that facilitators should be properly trained. However, there is a dearth of literature evaluating training programmes for critical incident debrief facilitators.

**What this study adds:**

- Professionals who attended a 5-day training programme based on critical incident stress debrief principles reported significantly higher confidence in supporting peers following incidents and facilitating post-incident structured team discussions after the training.
- Most participants reported that they would do things differently because of the training and that they found the training relevant, useful and engaging.
- Summative content analysis of qualitative feedback indicated that participants (i) believed the role plays were an important learning tool and (ii) thought it was important that the trainer was engaging. Some participants would have preferred in-person delivery.

**What impact this may have on practice or policy:**

- This is the first study to evaluate a training programme for post-incident discussion facilitators, and suggests the training was valued by participants.
- Organizations looking to implement post-incident discussions may benefit from employing a similar training programme, incorporating experiential role-play exercises.
- While online training delivery is preferred by some participants, others would prefer in-person delivery. Where possible organizations should aim to provide both options to prospective trainees.

## Introduction

Traumatic work events are common in clinical healthcare settings [1]. These can include sudden patient deaths [2], violence or aggression from patients [3] or involvement in adverse events, where patients are harmed in the course of care delivery [4]. Exposure to these events has increased since the onset of the COVID-19 pandemic, which has been characterized by inadequate material and human resources, high rates of patient mortality and challenging interpersonal dynamics in frontline healthcare settings [5].

Clinicians who are involved in such events can experience a range of negative outcomes including reduced job satisfaction and loss of confidence to symptoms of depression, anxiety and post-traumatic stress disorder (PTSD) [6,7]. When organizations do not respond supportively following these events, staff can feel isolated and abandoned, which can further exacerbate their mental distress [3].

Critical incident debriefs are one long-standing technique which have been used in clinical and emergency settings [1]. Distinct from learning-oriented debriefs [8], these draw together groups of staff who are united by a shared experience of a traumatic clinical event for a structured discussion delivered by a trained facilitator [9]. They are an occupational health tool to normalize and validate acute stress reactions [10]. There are three main team debriefing approaches: critical incident stress debriefing (CISD) [11], trauma risk management [12] and psychological debriefing [13]. These approaches build upon the natural tendency for workers who are exposed to traumatic events to engage in informal debriefing conversations with their colleagues [3].

While practitioners have used debriefs for years, research has produced mixed findings. One early review found that psychological debriefing did not reduce the risk of PTSD in trauma-exposed individuals, and included one study in which debriefing increased the risk of PTSD [14]. The subsequently published National Institute of Clinical Health and Care Excellence (NICE) guidelines recommended that psychological debriefing should not be used to prevent PTSD [15]. However, many studies included within this review and the NICE analysis have been criticized on multiple aspects, including that they: (i) used debriefing one-to-one rather than in a group; and (ii) used debriefing with individuals exposed to personal physical traumas rather than those exposed in a work capacity [10].

More recent research indicates that when used as an occupational health tool, as originally intended, debriefing is beneficial. A recent meta-ethnographic systematic review suggested that debriefs were consistently evaluated by trauma-exposed workers as helpful [1]. The authors concluded that while team debriefs cannot prevent PTSD, they offer benefits in helping workers to integrate their experiences, normalize their reactions and promote recovery [1]. Similarly, a recent systematic review and meta-analysis of debriefing in group settings concluded that while debriefing did not reduce risk of PTSD symptoms, there was evidence it was associated with reduced anxiety and problematic alcohol use [16].

These studies indicate that critical incident debriefs offer psychological benefits for participants such as catharsis, peer support and distress normalization [9]. Accordingly, post-incident team debriefing is now frequently recommended by contemporary healthcare researchers [17–20], provided that certain conditions are adhered to, such as optional participation and the use of a constructive approach [18].

Of the recommendations discussed by researchers for appropriate debriefing, the most frequently highlighted is that facilitators should be properly trained [10,17]. However, there is a dearth of literature evaluating training programmes for critical incident debrief facilitators. Studies are needed to understand (i) which training approaches are associated with increases in facilitator confidence; and (ii) how training approaches can be improved to make them more engaging, informative and accessible to participants. It is important to understand these questions in a post-COVID-19 pandemic context for two main reasons. First, the pandemic has caused healthcare practitioners unprecedented levels of stress and burnout, highlighting the need for better staff support interventions [5]. Second, the pandemic has increased the use of online platforms to deliver training interventions, but it is unclear how use of these platforms impacts trainees’ learning experiences.

The present study addressed this gap by conducting the first evaluation of a training programme for debrief facilitators. This programme trained facilitators in critical incident stress management (CISM), which builds on and incorporates training in the CISD approach [11]. Please see Appendix 1 (available as Supplementary data at *Occupational Medicine* Online) for more information.

The programme was delivered to staff and volunteers working in the UK National Healthcare Service (NHS), social care sector or voluntary, community and social enterprise (VCSE) sector entirely remotely, via an online platform over five working days. It was organized and commissioned by the regional Staff Wellbeing Hub. This is a one of 40 such Hubs in the UK which have been commissioned in response to the COVID-19 pandemic. The Hubs coordinate mental health support for staff in all these sectors. The evaluation addressed the following research questions:

1. Was participating in the training programme associated with increases in confidence in supporting colleagues and facilitating discussions after critical incidents?
2. How was the programme received by participants, and what possible improvements did they identify?

## Methods

The study utilized a single-arm, before-after design, which is useful for measuring a variable before and after the introduction of an intervention [21]. Data were collected via an online survey at two time points approximately 1 month apart: (i) prior to training (ii) directly after training. Data were collected from four training cohorts between October 2021 and January 2022. The project was conducted as part of a larger programme of research [22] which received ethical approval from the School of Psychology, University of Leeds ethics committee (Ref: PSYC-277; Approval date: 08/06/2021).

Participants responded to advertisements circulated via their regional Staff Wellbeing Hub. They could express interest if they had a local partner with whom they could deliver subsequent debriefs and managerial support to participate. After they expressed interest, the Staff Wellbeing Hub selected applicants to gain a representative spread of the local workforce and geographical region. Altogether, four 5-day training programmes were delivered by two different providers. Once they had been allocated to a training programme, participants were invited to participate in the research study via e-mail from the programme organizer. If they chose to participate, they provided informed consent and completed the online survey prior to the course (baseline). They then responded to the second survey on completion of the training (follow-up).

Demographic data pertaining to participants’ occupational group, gender, ethnicity and age (measured in 10-year age categories) were collected. Our primary outcome variable was confidence in debriefing facilitation skills. No appropriate and relevant scale could be identified to assess this, so we created two items for the purposes of the study. The first was ‘I am confident I would know how to support my colleagues if we experienced a critical incident in our team or unit’; the second was ‘I am confident I could facilitate a team discussion after a critical incident in my workplace’. Items were scored on a 5-point Likert scale (where ‘1’ was ‘Strongly disagree’ and ‘5’ was ‘Strongly agree’) and summed to form one overall score for ‘Confidence’.

Feedback data pertaining to experiences of the training were collected. Four items had a 5-point Likert response scale (where ‘1’ was ‘Strongly disagree’ and ‘5’ was ‘Strongly agree’) (see Table 2). Four items had ‘yes/no’ response options and provided space for participants to expand on their responses using free text (see Table 2). An additional item was included which allowed for free-text responses addressing the statement: ‘Now you have completed the training what additional support do you need from the hub to help you use the skills back in your organisation?’. A final item was included to gather general feedback responses in a free-text format: ‘Please use this space to add any additional comments about the training’. This survey is based on one used in a previous similar evaluation [23].

All quantitative analyses were completed using IBM SPSS software version 24. Descriptive statistics were used to describe the sample (gender, age group and ethnicity). To investigate whether the training was associated with increases in Confidence, we employed a paired-samples *t*-test. A *P*-value of ≤0.05 was considered statistically significant [24,25]. Finally, descriptive statistics were used to analyse responses to the feedback survey items, and a summative content analysis was used to analyse the free-text responses [26]. A summative analysis was chosen for its versatility in analysing text of different lengths and complexity. This is useful when analysing free-text box entries as the quality, length and content of these entries can vary. A summative content analysis allows for the researcher/s to generate a range of insights into the texts while preserving the integrity of the speaker’s voice and illuminating how words are used within the context of which they are spoken or written [26].

## Results

All 45 participants who received the training (32 females; 12 males; and 1 with unspecified gender; Table 1) responded to the baseline questionnaire or at the follow-up time point. Thirty-six participants provided responses at both the baseline and follow-up time points (Table 1).

**Table 1.** Descriptive information

		All participants (%)	Participants who completed both time points (%)	Participants who completed only one time point (%)
Participant gender, *N* (%)	Male	12 (27)	8 (22)	4 (44)
	Female	32 (71)	28 (78)	4 (44)
	Unknown/prefer not to say	1 (2)	0 (0)	1 (11)
Participant age group, *N* (%)	21–30	4 (9)	3 (8)	1 (11)
	31–40	12 (27)	11 (31)	1 (11)
	41–50	17 (38)	15 (42)	2 (22)
	51–60	10 (22)	7 (19)	3 (33)
	61–70	0 (0)	0 (0)	0 (0)
	71–80	1 (2)	0 (0)	1 (11)
	Unknown/prefer not to say	1 (2)	0 (0)	1 (11)
Ethnicity group, *N* (%)	White British	39 (87)	32 (89)	7 (78)
	British (unspecified)	1 (2)	1 (3)	0 (0)
	Asian British	1 (2)	1 (3)	0 (0)
	White European	2 (4)	1 (3)	0 (0)
	Mixed (White Asian)	1 (2)	0 (0)	1 (11)
	Unknown/prefer not to say	1 (4)	1 (3)	1 (11)
Job role categories, *N* (%)	Clinical psychologist	13 (29)	12 (33)	1 (11)
	Consultant or doctor	3 (7)	3 (8)	0 (0)
	Nurse or midwife	6 (13)	5 (14)	1 (11)
	Senior management	7 (16)	4 (11)	3 (33)
	Mental health specialist	6 (13)	5 (14)	1 (11)
	Support worker	2 (4)	2 (5)	0 (0)
	Paramedic or resuscitation officer	1 (2)	1 (3)	0 (0)
	Sister or matron	1 (2)	0 (0)	1 (11)
	Chaplaincy	1 (2)	1 (3)	0 (0)
	Occupational therapy	1 (2)	1 (3)	0 (0)
	Patient safety and improvement	2 (4)	2 (5)	0 (0)
	Unknown/prefer not to say	2 (4)	0 (0)	2 (22)
Participants’ work sector, *N* (%)	NHS	36 (80)	32 (89)	4 (44)
	VCSE	2 (4)	0 (0)	2 (22)
	Social care	3 (7)	2 (5)	1 (11)
	Other	2 (4)	2 (5)	0 (0)
	Unknown/prefer not to say	2 (4)	0 (0)	2 (22)

A statistically significant increase in Confidence, from before training to after training was found (*t*(35) = −6.77, *P* < 0.001). Scores increased from a mean of *M* = 6.64 (SD = 1.50) before training to a mean of *M* = 8.61 (SD = 1.23) after attending the debrief training.

The feedback from the debrief training was largely positive (Table 2). Most participants either strongly agreed or agreed the training was relevant for their role and that the skills they learned during the training were useful for their organization. Most participants also agreed or strongly agreed there was adequate time to cover the material and that they found the training engaging.

**Table 2.** Feedback following the training

Item	Strongly disagree (%)	Disagree (%)	Neither agree/disagree (%)	Agree (%)	Strongly agree (%)	Missing (%)
The training was relevant to my role	0 (0)	0 (0)	1 (2)	13 (29)	25 (56)	6 (13)
I learned skills in the training which will be useful for my organization	0 (0)	0 (0)	0 (0)	11 (24)	28 (62)	6 (13)
There was adequate time to cover the material	0 (0)	1 (2)	0 (0)	12 (27)	26 (58)	6 (13)
I found the training engaging	0 (0)	1 (2)	0 (0)	9 (20)	29 (64)	6 (13)
	Yes	No	Missing			
Were there any aspects of the training you did not find useful	5 (11)	33 (73)	7 (16)			
Is there anything else you would have liked to see in the training which was not included?	6 (13)	32 (71)	7 (16)			
If you were involved in a critical incident, would you do anything differently as a result of attending this training?	28 (62)	10 (22)	7 (16)			
Would you recommend the training to other staff or volunteers?	38 (84)	0 (0)	7 (16)			

A minority of participants indicated there were aspects of the training that they did not find useful (*N* = 5, 11%). The free-text responses were primarily directed towards the disadvantages of the virtual learning environment, e.g. screen fatigue, but also included a dislike for role-play learning and too much focus on health and safety information.

A minority of participants indicated that they would have liked to see additions to the training (*N* = 6, 13%). Their free-text responses focused on receiving more guidance and support on how to deliver debriefs in their sector (i.e. within the NHS, VCSE etc.), understanding the impact of trauma in an individual and supporting debriefs in complex cases.

Most participants indicated they would respond differently to a critical incident because of the training (*n* = 28, 74%). Participants’ responses indicated they would respond with a better knowledge and understanding of the effects of trauma following a critical incident, they would utilize skills developed in the training (e.g. follow a structured process) and that they would feel empowered to request a debrief or signpost colleagues to appropriate support services.

Most participants reported that they would recommend the training to colleagues (*N* = 38, 84%). Participants were also asked to indicate what additional support they would require to implement the training in their organization. A total of *n* = 25 (69%) participants responded and indicated a desire and preference for: peer support from other debrief facilitators; regular training to refresh their knowledge and to help them with ongoing skill development; support with implementing a strategy for facilitating debriefs in their organizations; to be informed about the range of support services available to them.

Free-text responses regarding the training were collated into key themes which were then categorized by frequency of remarks. Participants indicated that their experience of training focused on two key themes; (1) knowledge and skills of their training facilitator (*N* = 6), and (2) facilitation and delivery of training, including the use of role-play techniques (*N* = 11). Participants indicated the usefulness of role play as a learning device despite their general dislike for the technique (Table 3).

**Table 3.** Results from the summative content analysis

Theme	Description	Quotes
1: Knowledge and skills of their training facilitator	Role play is a challenging but useful device for learning	‘Never thought the day would come when I said that I enjoyed the role play elements…. But I did. Really powerful learning experience’. ‘It was great, really informative. Role plays are challenging but necessary I think’.
2: Facilitation and delivery of training	An engaging facilitator is key to a positive learning experience	‘The trainers were very personable, knowledgeable and able to make the group feel at ease discussing difficult topics’. ‘[Trainer name] has been amazing – difficult to do on this platform but she managed the pace and content so well. Very supportive and engaging’.

Participants were also keen to express how important their training facilitator was in providing a supportive and encouraging learning environment. Participants indicated that their facilitators, knowledge, experience and communication skills were paramount in providing a valuable and productive training experience (Table 3).

## Discussion

This is the first evaluation of a training programme for critical incident debriefers. Participating in the training programme was associated with increases in confidence in supporting colleagues and facilitating discussions after critical incidents. The training feedback indicated that participants found it engaging and relevant for their role, and that they believed it would offer benefits for their organization. A minority of participants identified possible improvements that could be made. These included a reduction in focus on health and safety information and a move to in-person delivery, rather than online delivery.

A strength of the study was its use of a mixed-methods design, which enabled the reporting of quantitative outcome data alongside the collection of data which could be used to inform the development of future facilitator training interventions. This is particularly important given the novelty of this study; with so little information currently available, qualitative data are crucial for informing future practice and research. The study also benefited from the use of an online data collection design. By avoiding paper data collection by the training facilitator, this approach protected participants’ anonymity within the study and reduced the risk of demand characteristics.

The first limitation of the study was its use of an uncontrolled design which means it is not possible to conclude that any significant changes in outcome variables can be attributed to the intervention. A second limitation was the use of subjective response measures, which prevent conclusions regarding objective improvements being drawn. Third, this training programme was delivered entirely remotely, so findings cannot necessarily be generalized to in-person courses which may be delivered in future. Fourth, the participants were of disproportionately White ethnicity, which is not consistent with the wider ethnic diversity of health, social care and VCSE employees.

Several studies have investigated the outcomes of critical incident stress debriefs [1,16]. These indicate that debriefing should not be used as a PTSD prevention intervention for individuals [14,15], but it is a useful occupational health tool for groups exposed to traumatic work events [1,10,16]. However, we are aware of no existing studies which have investigated training interventions for CISD. As this form of debriefing is frequently recommended by practitioners [19,20,27,28], this lack of information regarding training approaches is concerning, particularly given strong recommendations that facilitators should be trained [10,17]. At present, organizations looking to implement trauma incident pathways involving debriefing lack guidance on how best to do this and may use inefficient or ineffective training approaches as a result. The current study addresses this gap by presenting the first evaluation of a debriefing training programme. This study indicates that this intervention was associated with significant increases in confidence and overall positive feedback. It also highlighted that (i) including role plays and (ii) using an engaging facilitator were important ingredients of the training programme. Programmes could be improved by being including in-person options. They could also be improved by moderating the amount of health and safety information which is included.

This lack of evidence regarding CISD is surprising when considered in relation to training for other non-clinical skills in healthcare. For example, a review of training courses for breaking bad news interventions identified 17 controlled studies of training interventions in physicians alone [29]. Similarly, a systematic review of randomized controlled trials evaluating resilience training interventions in healthcare professionals identified 39 eligible studies [30]. This limited literature could reflect the lack of clarity around the use of debriefing which was generated following the 2002 Cochrane review and subsequent NICE analysis [14,15]. However, it is now clear that while it cannot be used to prevent PTSD, debriefing offers multiple benefits, including reduced anxiety [16]. The present study provides initial evidence which can be used to support organizations looking to implement support pathways for groups of staff involved in traumatic work events. This has become an organizational priority following the onset of the COVID-19 pandemic, which caused an increase in these types of events [5].

The present findings provide support for the training programme evaluated in the present study. This programme trained participants in CISM, which builds on and incorporates the CISD approach [11]. Participants found a 5-day programme an acceptable length and participants from multiple different disciplines and health care settings believed the training programme had relevance for their discipline. These findings also highlight that organizations seeking to implement such training programmes should ensure that this (i) incorporates role play and (ii) is delivered by an enthusiastic, engaging and well-informed facilitator. However, organizations should consider the mode of delivery for this training; while online delivery may be acceptable and accessible for many participants, some will prefer in-person options.

This study is the first the authors are aware of which evaluates a training intervention for critical incident debrief facilitators. We have several recommendations for future research. First, studies should examine programmes which differ in length and delivery style and should use controlled designs. Second, studies should use behavioural measurements to establish whether the training produces objective improvements in debriefing skills. Studies should also explore debrief recipients’ experiences of being debriefed and test whether these are affected by the level and type of training a facilitator has received, and whether they are a qualified mental health professional or peer supporter by background. Third, studies may explore the effects of past incident exposure on training perceptions and responses, to see whether this impacts engagement or learning. Fourth, studies should use longer follow-ups to see whether perceived improvements are maintained over time.

Critical incident debriefing is a useful occupational health tool to support staff following traumatic work events [1]. It is frequently recommended that debrief facilitators should be trained, but there is a dearth of evidence evaluating relevant training programmes. The present study provides evidence for a 5-day training programme which aimed to equip multidisciplinary healthcare staff to act as peer supporters and debrief facilitators and found that this received mostly positive feedback. Organizations looking to implement debrief facilitator training programmes as part of staff support pathways should ensure that such programmes are delivered by informed, engaging facilitators and that these include role plays.

## Supplementary Material

kqac125_suppl_Supplementary_Appendix
