# Supplementary material for: Evaluation of a training programme for critical incident debrief facilitators
Source: Occup Med (Lond). 2022 Dec 14;73(2):103–8. doi: 10.1093/occmed/kqac125 (PMC10016050; doi:10.1093/occmed/kqac125)
Supplement: kqac125_suppl_Supplementary_Appendix [file kqac125_suppl_supplementary_appendix.docx]

| Appendix 1: Training programme information | |
| --- | --- |
| Aims | To equip participants in the full package of Critical Incident Stress Management (CISM) skills |
| Components | Modules within the training programme covered the following topics:   - Learning skills to facilitate and deliver CISDs - Understanding reactions to trauma and identifying symptoms of Post-Traumatic Stress Disorder (PTSD) - Understanding the literature base for CISM and other psychological debrief interventions - Learning about relevant NICE guidelines - Learning skills for conducting ‘hot’ debriefs and strategies for defusing emotions in the immediate aftermath of incidents. |
| Group mix and size | Groups included delegates from various professional backgrounds; around 10 participants were included in each training cohort but some exercises used ‘breakout rooms’ to facilitator smaller group work sessions. |
| Modality | Entirely remote delivery, via video-platform. |
| Techniques | - Didactic teaching - Group discussions - Small group exercises - Role play and reflection |
| Conceptual approach | The CISM training considered CISD as one part of a jigsaw of skills needed to support professionals who have been involved in incidents. The 7-stage model (Mitchell & Everly, 1996) was used to structure the approach to debriefing. The 7 stages are:   1. **Pre-crisis preparation**: education in managing and coping with stress; training in ways to mitigate incidents for both individuals and organisations 2. **Disaster or large-scale incident support programmes**: delivering large community meetings to provide information; advising staff 3. **Defusing:** a discussion provided within hours of the incident to small groups, using a 3-phase structure. Aims to assess, triage and mitigate acute symptoms 4. **Critical Incident Stress Debriefing (CISD):** a discussion provided 1 to 10 days after the incident to small groups, using a 7-phase structure. Aims to assess, triage and mitigate acute symptoms. If possible, the discussion will provide a sense of psychological ‘closure’ about the incident. 5. **One-on-one crisis intervention/counselling or psychological support:** where necessary, one-to-one support will be provided throughout the incident. 6. **Family crisis intervention**: Consultation will be provided to families or organisations as appropriate. 7. **Referral and follow up**: Where acute distress and trauma symptoms warrant referral and further treatment, mechanisms for assessment and treatment will be made available. |
| Facilitator knowledge and background | One of two facilitators delivered the training programme. Both were mental health professionals (a Cognitive Behaviour Therapist and Psychotherapist) who were experienced in working with trauma and providing CISM training to National Health Service (NHS) staff. |

Mitchell, J.T. & Everly, 0.5. (1996 ). Critical Incident Stress Debriefing: An Operations Manual. Ellicott City , MD : Chevron.
